# InfPolyn, a Nonparametric Bayesian Characterization for Composition-Dependent Interdiffusion Coefficients

**DOI:** 10.3390/ma14133635

**Published:** 2021-06-29

**Authors:** Wei W. Xing, Ming Cheng, Kaiming Cheng, Wei Zhang, Peng Wang

**Affiliations:** 1 School of Integrated Circuit Science and Engineering, Beihang University, Beijing 100191, China; wxing@buaa.edu.cn; 2 Department of Civil and Environmental Engineering, University of Illinois Urbana-Champaign, Urbana, IL 61801-2352, USA; ming6@illinois.edu; 3 Shandong Key Laboratory for High Strength Lightweight Metallic Materials, Advanced Materials Institute, Qilu University of Technology (Shandong Academy of Sciences), Jinan 250014, China; 4 Sino-French Engineer School, Beihang University, Beijing 100191, China; wei.zhang@buaa.edu.cn; 5 Beijing Advanced Innovation Center for Big Data and Brain Computing, Beihang University, Beijing 100191, China

**Keywords:** Gaussian process, nonparametric Bayesian, interdiffusion coefficient, Boltzmann–Matano analysis

## Abstract

Composition-dependent interdiffusion coefficients are key parameters in many physical processes. However, finding such coefficients for a system with few components is challenging due to the underdetermination of the governing diffusion equations, the lack of data in practice, and the unknown parametric form of the interdiffusion coefficients. In this work, we propose InfPolyn, Infinite Polynomial, a novel statistical framework to characterize the component-dependent interdiffusion coefficients. Our model is a generalization of the commonly used polynomial fitting method with extended model capacity and flexibility and it is combined with the numerical inversion-based Boltzmann–Matano method for the interdiffusion coefficient estimations. We assess InfPolyn on ternary and quaternary systems with predefined polynomial, exponential, and sinusoidal interdiffusion coefficients. The experiments show that InfPolyn outperforms the competitors, the SOTA numerical inversion-based Boltzmann–Matano methods, with a large margin in terms of relative error (10× more accurate). Its performance is also consistent and stable, whereas the number of samples required remains small.

## 1. Introduction

In many industrial processes that involve diffusion, e.g., alloy solidification, heat treatment, coating and electric packaging, the characterization of composition-dependent interdiffusion coefficients is a crucial task, as it quantifies a diffusion process clearly. The classic approach is based on Boltzmann–Matano analysis [1,2] which transforms the diffusion system into a linear system of equations. However, the Boltzmann–Matano analysis is only applicable to a binary system and becomes problematic for a system with more than three components, as it generates an under-determined system of equations that mathematically does not yield a unique solution. To address such a challenge, a number of methods have been developed over the years. Kirkaldy et al. [3] introduced the Kirkaldy–Matano method and provided extra equations to the linear system by adding additional *M* diffusion paths with intersection points. Although the results have been shown to be accurate, this method cannot generalize well to a multi-component system because the difficulty in experimentally generating intersection points grows drastically with the number of components M+1 [4]. Alternatively, methods based on one diffusion couple were proposed. Dayananda and Sohn [5] suggested integrating over certain composition ranges along the diffusion path to evaluate an average interdiffusion coefficient. Cermak and Rothova [6] later extended this method by choosing an infinitely small integration interval. Nevertheless, as is pointed out by Cheng et al. [7], the integration approach can lead to ill-conditional problems. A pseudobinary approach is introduced by considering only two components diffused into the diffusion zone. This method takes advantage of its time independence in the first-order linear equations and thus is very efficient when the pseudobinary condition is strictly satisfied in experiments. In practice, such experimental conditions may be difficult to meet, and in addition, for a multi-component system with a limited number of experimental samples, the linear equations are not capable of eliminating the extra solutions [8,9]. Separately, Zhang and Zhao [10] suggested a forward-simulation approach by iteratively optimizing the interdiffusion coefficients with repeated forward-simulations, similar to the classic inference approach for inverse problems. Although such a method is shown to be accurate and stable, it incurs an overwhelming computational cost because each iteration requires a complete diffusion simulation with a fine spatial-temporal grid.

Another branch of the one-diffusion-couple method lies in assuming a polynomial functional form for the interdiffusivities. Ideally, with a proper design of the polynomial function, one can compute the coefficients of the polynomial functions to estimate the interdiffusion using a numerical inverse method [11]. This numerical inverse approach is adopted by Chen et al. [4] to include the atomic mobility [12] to study the diffusion in the solution phase of a multicomponent system. To improve the efficiency of the numerical inverse method, Cheng et al. [13] recast the original parabolic inverse problem [11] as a linear multi-objective optimization to improve computation efficiency while maintaining similar accuracy. The optimization algorithm places weak limits on the experimental samples and is applied to interdiffusivities of solid solution as well as various alloy systems [14,15]. This approach was recently improved by Qin et al. [16], who suggest solving an underdetermined linear system using compress sensing, a popular regularization technique, to increases stability against high order polynomial functions. However, the $L^{1}$ penalty imposed by compress sensing may introduce inappropriate prior assumptions, leading to inferior overall performance. We will see this issue in detail in the later experiment section.

Despite the notable performance and the popularity of the polynomial functional interdiffusion coefficient approaches, they share a fatal issue—how does one design the polynomial functions? Considering a quadternary system (M=3), we have 3×3 polynomial functions requiring careful designs; modifying one function will affect the results of the other two. The challenge grows quadratically with the number *M*. Without proper design and repeated validations, the polynomial approach will lead to overfitting or underfitting, making this approach infeasible in practice.

One way to resolve this challenge is to use a complicated enough model with many polynomial terms and utilize classic Bayesian inference techniques [17] to estimate the posterior of the polynomial coefficients. In particular, Girolami [18] proposed an interesting Markov chain Monte Carlo (MCMC) for nonlinear and complex differential equations where the fully analytic expressions for the posterior distribution do not exist, which is similar to our problem. Despite its elegance and great accuracy, an MCMC approach often suffers from slow convergence and poor mixing, making it less practical for complex applications. To improve inference efficiency, the approximate Bayesian computation (ABC) and their variations, e.g., MCMC ABC and sequential Monte Carlo ABC (SMC ABC) are put forth by Alahmadi et al. [19]. However, despite being accurate and easy to implement, these types of sampling methods do not scale well with the number of parameters to be inferred. With unknown polynomials, the large number of parameters makes such methods impractical even with the latest accelerated variations [20,21].

Recently, the Gaussian process (GP) [22] has been utilized in dealing with data that are generated from a system of differential equations. As a back-box regression model, GP is proposed for fast parameter posterior estimations with the derivative information of the differential equations even with partially observed data [23]. The explicit derivative information is further utilized to improve a general GP’s performance for data that are generated from differential equations [24]. The derivative in a given system of differential equations is further harnessed through a constraint manifold such that the derivatives of the Gaussian process must match an ordinary differential Equation (ODE) [25]. Despite their success, these works generally require explicitly known differential equations to work. Thus, they cannot directly be implemented for our problem.

A closely related work is [26], where GP is used as a generalization for a parametric function for binary images. However, their work cannot be directly implemented in our problem because our systems of equations will lead to a mixture of GPs that are augmented by the derivative of concentrations, whereas there is normally only one GP to estimate in most of the previous works [23,24,25,26,27].

To address the challenge of stable characterization of the interdiffusion coefficients, we introduce InfPolyn (Infinite Polynomial), a nonparametric Bayesian framework for the characterization of composition-dependent interdiffusion coefficients.

In particular, we first extend the general polynomial fitting method with an infinite number of polynomial terms. We then integrate out the polynomial coefficients with a Gaussian prior to derive a nonparametric functional form for the interdiffusion coefficients. To further improve our model with prior assumptions of an interdiffusion system, we introduce a diagonal-dominant prior for the functions of the interdiffusion coefficients. Unlike most Bayesian fitting problems, the interdiffusion coefficients are not known/observable to us. Thus, we introduce latent variables, the virtual ghost interdiffusion coefficients to address this issue. Finally, we derive a tractable joint likelihood function for model training. We compare InfPolyn with the state-of-the-art Matano-based numerically inverse methods and their variations. In ternary and quaternary systems with polynomial, exponential, and sinusoidal interdiffusion coefficients, InfPolyn shows a significant improvement over the competitors in terms of relative errors. In most of the experiments, our model shows an excellent performance with only 40 EPMA measurements, which is very desirable in practical interdiffusion coefficient estimations.

Essentially, InfPolyn is a functional estimation method tailored for the characterization of interdiffusion coefficients by imposing a mixture of the SOTA nonparametric models, GPs, and particular prior knowledge. Unlike the classic Bayesian inference approaches [18,19], InfPolyn does not require a time-consuming sampling process and is thus much more efficient. The highlights of this work for interdiffusion coefficient characterizations are as follows:

- InfPolyn does not require assumptions for the particular functional form of the interdiffusion coefficient; it is robust against overfitting and underfitting.
- InfPolyn does not require a significant number of training data.
- Prior knowledge of the interdiffusion system can be added easily in the framework of InfPolyn.

We hope the success of the nonparametric Bayesian framework can inspire more interesting applications in other interdiffusion coefficient estimation methods, e.g., the forward-simulation approach [10], in the material community. Thus, we publish our code and will maintain it as an open source toolbox on Github (https://github.com/wayXing/InfPolyn, accessed on 26 June 2021).

The rest of this paper is organized as follows. The interdiffusion coefficient estimation problem is introduced in Section 2, followed by a brief summary of the Matano–Boltzmann numerical inverse method with polynomial functions in Section 3. Our method is presented in Section 4, including the derivation, prior knowledge assumptions, and model training. The comparisons to the other SOTA methods through ternary and quandary systems are demonstrated in Section 5. Finally, Section 6 summarizes our work.

## 2. Statement of the Problem

We firstly formulate our problem mathematically as a foundation of this work. Consider a general one-dimensional diffusion system with (M+1) components. According to Fick’s second law [28], the diffusion process is fully characterized by

(1) $$\begin{array}{c} \frac{\partial c^{i}}{\partial t}=\nabla \left(\sum_{j=1}^{M}D_{ij}\nabla c^{j}\right),\;i=1,\cdots ,M, \end{array}$$

where ∇ is the partial derivative operator, $c^{i}$ is the concentration of *i* component (note that the concentration is a function of space and time $c^{i}\left(t,x\right)$); $D_{ij}$ is the interdiffusion coefficient w.r.t. the concentration gradient of component *j*. In many textbook examples, $D_{ij}$ is assumed constant, but in practice, $D_{ij}$ depends on the concentrations of all components $c={\left(c^{1},\cdots ,c^{M}\right)}^{T}$. Our goal is to find $D_{ij}\left(c\right)$ for all i,j=1,⋯,M with, ideally, a concentrations profile $C={\left(c{\left(t_{e},x_{1}\right)}^{T},\cdots ,c{\left(t_{e},x_{N}\right)}^{T}\right)}^{T}\in R^{N\times M}$ at some terminal time $t_{e}$ and spatial locations ${\left\{x_{n}\right\}}_{n=1}^{N}$, where *N* is the number of sampling points at different locations. To avoid clutter, we denote $c_{n}=c\left(t_{e},x_{n}\right)$. One may notice that an important factor, temperature, is not considered in the formulation. This is due to the general process of the experiment. To conduct the experiment and obtain the concentration profile, one first bonds two blocks of materials together and holds them at certain temperatures to activate interdiffusion at the initial interface. The annealing procedure may last from hours to days, depending on the speed of forming an interdiffusion zone wide enough for analysis. The temperature remains constant during the long-lasting annealing process except for the beginning and ending stages, which take short time. Thus, the temperature is considered constant for the interdiffusion coefficient characterizations. To fabricate just one diffusion couple, around 50–100 sample points are often selected in a line parallel to the direction of element diffusion within the interdiffusion zone. Each sample point is analyzed through electron probe micro-analysis (EPMA), which requires several minutes for the equipment to detect the concentrations. As a result, the experiment is time-consuming, and only a small amount of samples, i.e., small *N*, can be provided.

## 3. Boltzmann–Matano Polynomial Interdiffusion Coefficients

We follow the original work of the Boltzmann–Matano method [2], which is widely used to extract concentration-dependent interdiffusion coefficient $\left\{D_{ij}\right\}$ from experimental concentration profiles. The Boltzmann–Matano method first integrates Fick’s law of diffusion (1) in time to obtain the following system,

(2) $$\begin{array}{c} \frac{1}{2t}\int_{0}^{c^{i}}\left(x-x_{0}\right)dc^{i}=-\sum_{j=1}^{M}D_{ij}\nabla c^{j},\;i=1,\cdots ,M, \end{array}$$

where $c^{i}$ denotes the terminal concentration of i components, $\nabla c_{j}$ is the concentration gradient, and $x_{0}$ is the known Matano plane, defined by

(3) $$\begin{array}{c} \int_{-\infty }^{x_{0}}\left(1-c(x)\right)dx=\int_{x_{0}}^{+\infty }c\left(x\right)dx. \end{array}$$

For a binary system, i.e., M+1=2, there is only one composition-dependent interdiffusion coefficient $D_{11}$ to determine with one diffusion couple. Based on Equation (2), we can can directly compute $D_{11}\left(c_{n}\right)$ for n=1,⋯,N and then use any curve-fitting method to characterize the function of $D_{11}\left(c\right)$. For a ternary system, i.e., M=2, we need to determine $D_{ij}\left(c\right)$ for i={1,2} and j={1,2}. For each sample $c_{n}$, we can write only two equations whereas there are four unknown parameters. This is an underdetermined system of equations to solve and will lead to multiple solutions. An effective and efficient solution is to assume a continuous function of interdiffusivity in a polynomial form, e.g., an independent quadratic form,

(4) $$D_{ij}\left(c\right)=w_{ij}^{\left(0\right)}+\sum_{i=1}^{M}\left(w_{ij}^{\left(i\right)}c^{i}+w_{ij}^{\left(M+i\right)}{\left(c^{i}\right)}^{2}\right),$$

where *w* is the weight coefficient in the polynomial function. Denote the flux of the L.H.S. of Equation (2) as *u*: we have $u^{i}=\left(u_{1}^{i},\cdots ,u_{N}^{i}\right)$, where $u_{n}^{i}=\int_{0}^{c^{i}\left(x_{n}\right)}\left(x-x_{0}\right)dc^{i}/2t$. Estimation of $D_{ij}$ for j=1,⋯,M can then be computed by solving the system of equation

(5) $$u_{n}^{i}=-\sum_{j=1}^{M}D_{ij}\left(c_{n}\right)\nabla c_{n}^{j},$$

where $D_{ik}\left(c_{n}\right)$ is the polynomial function fully determined by its weight coefficients given a particular functional form and $c_{n}$. All weight coefficients $W=\{w_{ij}^{k}\}$ in the polynomial functions can be computed by solving the optimization problem,

(6) $$\underset{W}{\mathrm{argmin}}\sum_{n=1}^{N}{∥u_{n}^{i}+\sum_{j=1}^{M}D_{ij}\left(c_{n}\right)\nabla c_{n}^{j}∥}^{2},$$

where ${∥\cdot ∥}^{2}$ denotes the $L^{2}$ norm, which can be replaced with other norms.

**Remark** **1.** *Since the estimation of $D_{ij}\left(c\right)$ for each i=1,⋯,M only depends on $u^{i}$ and is computed independently, we omit the index i and reformulate the Matano–Boltzmann method with polynomial interdiffusion coefficients to avoid clutter,*

(7) $$\underset{W}{\mathrm{argmin}}\sum_{n=1}^{N}{∥u_{n}+\sum_{j=1}^{M}d_{n}^{j}\nabla c_{n}^{j}∥}^{2}=\underset{W}{\mathrm{argmin}}\sum_{n=1}^{N}{∥u_{n}+\nabla c_{n}^{T}d_{n}∥}^{2},$$

*where* $u_{n}$ *is the flux for any arbitrary component, and* $\nabla c_{n}^{j}$ *is the concentration gradient for j component, both of which are computed from the profile* C *;* $d^{j}\left(c_{n}\right)$ *is the j column of any arbitrary row of* $D_{ij}\left(c\right)$ *that matches the chosen flux at concentration* $c_{n}$ *;* $d_{n}={\left(d^{1}\left(c_{n}\right),\cdots ,d^{M}\left(c_{n}\right)\right)}^{T}$ *is the collection. We aim to reveal* $d^{j}\left(c\right)$ *for* j=1,⋯,M *.*

### Optimization for Polynomial Fitting

Equation (7) is a convex optimization problem provided that we have N≥3(K+1)M EPMA samples and we use a *K*-order polynomial function of Equation (4) for all $d^{j}\left(c\right)$; the closed-form solution is presented in the Appendix A. This is certainly impractical for large *M* and/or *K*. In this case, regularization techniques, e.g., $L^{2}$-norm minimization or compress sensing, can be implemented to solve such an underdetermined system. The polynomial fitting approach with regularization is efficient in terms of computational time, space complexity, and implementation simplicity, thanks to many excellent software solutions, e.g., $l_{1}$-magic, *SPGL1*, and *SeDuMi* [29,30,31].

## 4. InfPolyn for Interdiffusion Coefficients

The challenge of the discussed polynomial based approach is the lack of guidelines on how to build the model, i.e., the selection of the order of the polynomial and the polynomial form. It is unclear how many polynomial terms are needed for each diffusion coefficient such that the model is not overfitting or underfitting. Although regularization techniques [16] can be implemented, the underlying assumptions of regularization are unclear, which can lead to unexpected performance. We need a systematic way to specify the diffusion coefficients with correct prior knowledge in order to achieve better results. To this end, we propose a nonparametric Bayesian approach that is flexible enough to capture the complex nonlinear relation while restricting itself from overfitting the data by integrating all possible solutions.

### 4.1. Infinite Order Polynomial Model

To start with, we write the polynomial regression, e.g., Equation (4), in a compact form,

(8) $$d^{j}\left(c\right)=w_{j}^{T}\phi_{j}\left(c\right)+\beta_{j},$$

where the polynomial terms are denoted compactly as $\phi_{j}\left(c\right)={\left(c^{1},{\left(c^{1}\right)}^{2},\cdots ,c^{2},{\left(c^{2}\right)}^{2},\cdots \right)}^{T}$, where $\phi_{j}\left(\cdot \right)$ is the predefined feature mapping that encodes the the polynomial functional form. Essentially, we can project the concentration c onto an r–dimensional feature space using an arbitrary mapping $\phi_{j}\left(c\right)\in R^{r}$. Note that the constant term can also be absorbed into the feature mapping by setting the first element as 1. In the linear model case, the feature mapping is simply $\phi_{j}\left(c\right)={\left(1,c^{T}\right)}^{T}$.

Obviously, this polynomial approach is only accurate and stable when we roughly know the functional form of $\phi_{j}\left(c\right)$. Furthermore, it requires a large number of parameters $\left\{w_{j},\beta_{j}\right\}$ to be estimated. Rather than estimating the weight parameters, we consider a matrix Gaussian prior for the weight vector $w_{j}$,

(9) $$w_{j}∼N\left(0,\Omega_{j}\right)=\frac{1}{\sqrt{{\left(2\pi \right)}^{r}\left|\Omega_{j}\right|}}\exp\left(\frac{-w_{j}^{T}\Omega_{j}^{-1}w_{j}}{2}\right),$$

where, $\Omega_{j}\in R^{r\times r}$ indicates the correlation between the weight components. We then integrate out the weights and directly work with the marginal, which admits a closed-form solution,

(10) $$\begin{array}{cc} p(d^{j}|c) & =\int p\left(d^{j}|w_{j},c\right)p\left(w_{j}\right)dw_{j}\\ \; & =\int \left(w_{j}\phi \left(c\right)+\beta_{j},\right)N\left(w_{j}|0,\Omega_{j}\right)dw_{j}\\ \; & =N\left(\beta_{j},{\left(\phi_{j}\left(c\right)\right)}^{T}\Omega_{j}\phi_{j}\left(c\right)\right). \end{array}$$

This is also known as the Gaussian process (GP) [22]. If we use a countably infinite feature space, i.e., r→∞, we formally define a sum over infinite polynomial terms. Thus, we call our model InfPolyn, infinite polynomial. Our model now becomes a nonparametric model that contains no explicit parameters $w_{j}$. The model parameters are now encoded in ${\left(\phi_{j}\left(c\right)\right)}^{T}\Omega_{j}\phi_{j}\left(c\right)$, which indeed indicates a inner product in the the feature space spanned by $\phi_{j}\left(c\right)$.

### 4.2. Kernel Formulation

Note that $\Omega_{j}$ is p.s.d. by its definition, and we can encode the inner product using a compact function, i.e., $k_{j}\left(c,c^{\prime }\right)={\left(\phi_{j}\left(c\right)\right)}^{T}\Omega_{j}\phi_{j}\left(c\right)$. This is known as the kernel trick, which works by replacing the explicit feature mapping and covariance with a kernel function $k_{j}\left(c,c^{\prime }\right)$ to indicate an inner product in the feature space. Different kernels can capture different functional features. For instance, a periodic kernel can capture periodic functions such as sinusoidal functions. If we do not know the explicit form of the kernel function, which is true in most cases, the automatic relevance determination (ARD) kernel,

(11) $$k_{j}\left(c,c^{\prime }\right)=\theta_{j0}\exp\left(-{\left(c-c^{\prime }\right)}^{T}{\left(I⊙({\tilde{\theta }}_{j}^{T}{\tilde{\theta }}_{j})\right)}^{-1}\left(c-c^{\prime }\right)\right),$$

is commonly adopted as it generally provides good performance in most cases, especially in regression problems [22]. In this formulation, ⊙ denotes the Hadamard product, I is an identity matrix, $\theta_{j0}$ is the scaling factor for the kernel function, and ${\tilde{\theta }}_{j}\in R^{M\times 1}$ is a vector with scaling factors for each input components, i.e., the concentrations of different elements. We denote $\theta_{j}={\left(\theta_{j0},{\tilde{\theta }}_{j}^{T}\right)}^{T}$ for clarity. These parameters $\theta_{j}$ are known as the hyperparameters because they control the random process (10) statistically rather than in a determinant way (e.g., the aforementioned polynomial fitting). In this work, we use the ARD kernel throughout unless stated otherwise.

### 4.3. Ghost Interdiffusion Coefficients

Given that $d_{j}\left(c\right)$ is a Gaussian process as stated in Equation (10), any number of observations form a joint Gaussian distribution, based on which a closed-form likelihood can be easily calculated. Unfortunately, unlike the classic regression problems, we do not have any direct observations of $d_{j}\left(c\right)$, and we cannot directly obtain the optimized hyperparameters $\left\{\theta_{j},\beta_{j}\right\}$. To resolve this problem, we borrow the pseudo-inducing points idea [32] and introduce a set of virtual ghost interdiffusion coefficients, ${\left\{h_{jg}=d_{j}\left(z_{jg}\right)\right\}}_{g=1}^{G}$, that are sampled from the function $d_{j}\left(c\right)$ for virtual concentrations ${\left\{z_{jg}\right\}}_{g=1}^{G}$. These latent variables must form a joint Gaussian distribution (because they are sampled from a Gaussian process of (10)),

(12) $$h_{j}∼N\left(\beta_{j}1,K_{j}\right),$$

where $h_{j}={\left(h_{j1},\cdots ,h_{jG}\right)}^{T}$ is the collection of the ghost interdiffusion coefficients and ${\left[K_{j}\right]}_{gg^{\prime }}=k_{j}\left(z_{jg},z_{jg^{\prime }}\right)$ is the covariance matrix computed through the kernel function and the latent locations $Z_{j}={\left\{z_{jg}\right\}}_{g=1}^{G}$. Normally, **h**_*j*_ and $Z_{j}$ are latent variables that need to be integrated out during the model training and predictions.

### 4.4. Diagonal-Dominating Prior

Following Occam’s razor, if the dominant diagonal diffusion coefficients $D_{ii}\left(c\right)$ for i=1,⋯,M can fully explain the diffusion process, it is reasonable to suppress the non-diagonal diffusion coefficients $D_{ij}\left(c\right)$ for i≠j to encourage a simpler model. To inject this preference of model, we design a special Laplace prior for the mean value for each Gaussian process of (12),

(13) $$\beta_{j}∼\mathrm{Laplace}\left(0.01^{\left(1-\delta (i,j)\right)},0.1\right),$$

where δ(·,·) is the delta function and *i* is the row that matches the choice of $d^{j}\left(c\right)$. We use a Laplace prior rather than a Gaussian prior to encourage sparsity of the diffusion concentration for non-diagonal locations. The particular prior parameters may be adjusted according to a different system to reflect our prior knowledge.

### 4.5. Joint Model Training

With each interdiffusion coefficient $d^{j}\left(c\right)$ fully specified previously, the observed flux $u_{n}$ can be recovered by

(14) $$u_{n}=f_{n}+\epsilon_{n}=\nabla c_{n}^{T}d\left(c_{n}\right)+\epsilon_{n},$$

where we use the noise term $\epsilon_{n}$ to capture the model inadequacy, uncertainty, and noise as a Gaussian distribution, $\epsilon_{n}∼N\left(0,\sigma^{2}\right)$, for the observed flux; $f_{n}$ denotes the unknown true flux.

Eventually, the last piece of this work is the the estimation of the posterior of all hyperparameters $\Theta ={\left\{\theta_{j}\right\}}_{j=1}^{M},B={\left\{\beta_{j}\right\}}_{j=1}^{M}$, $Z={\left\{Z_{j}\right\}}_{j=1}^{M}$, $H={\left\{h_{j}\right\}}_{j=1}^{M}$, and σ. Although MCMC can be directly implemented to compute all model parameter posteriors, the computational time is overwhelming considering the large number of hyperparameters and the efficiency of an MCMC procedure. Instead, we opt for the maximum a posterior (MAP) approach. The log posterior decomposes as the log likelihood and the prior information,

(15) $$\underset{\Theta ,B,Z,H,\sigma }{\mathrm{argmax}}\left(L(\Theta ,B,Z,H,\sigma )+\log p(B)\right),$$

where L(Θ,B,Z,H,σ)=logp(u) is the log likelihood of our model, which can be computed by comparing the predicted flux **f** and the observed flux **u**. More specifically, the log marginal likelihood can be computed by

(16) $$\begin{array}{c} \log p(u)=\log\int p(u|f)p(f)df \end{array}$$

To complete the integration in Equation (16), we first notice that

(17) $$p(u|f)=N\left(u|f,\sigma^{2}I\right)$$

is simply a Gaussian; p(f) is a mixture of *M* Gaussians, which is also Gaussian because

(18) $$\begin{array}{cc} p(f) & =\sum_{j=1}^{M}\nabla c^{T}d^{j}\left(c\right)=\sum_{j=1}^{M}\nabla c^{T}N\left(\mu_{j},Q_{j}\right)\\ \; & =\sum_{j=1}^{M}N\left(\nabla c^{j}⊙\mu_{j},\nabla c^{j}Q_{j}\nabla {\left(c^{j}\right)}^{T}\right)=N\left(\mu ,Q\right). \end{array}$$

In this equation, $\mu_{j}=\beta_{j}1+k_{j}{\left(K_{j}\right)}^{-1}\left(h_{j}-\beta_{j}1\right)$ is the predicted interdiffusion expectations for j; $Q_{j}={\hat{K}}_{j}-{\tilde{K}}_{j}K_{j}^{-1}{\tilde{K}}_{j}^{T}$ is the covariance matrix, with ${\left[{\tilde{K}}_{j}\right]}_{ng}=k_{j}\left(c_{n},z_{jg}\right)\in R^{N\times G}$ being the covariance between C and $Z_{j}$ and ${\left[{\hat{K}}_{j}\right]}_{nn^{\prime }}=k_{j}\left(c_{n},c_{n^{\prime }}\right)\in R^{N\times N}$ being the covariance for C. $\mu =\sum_{i=1}^{M}\mu_{i}⊙\nabla c^{i}$ is the joint expectations; $Q=\sum_{i=1}^{M}\nabla c^{j}Q_{j}\nabla {\left(c^{j}\right)}^{T}$ is the joint covariance matrix. Substituting Equations (17) and (18) into Equation (16) to derive the joint log likelihood, we get,

(19) $$\log p\left(u\right)=-\frac{1}{2}{\left(\mu -u\right)}^{T}{\left(Q+\sigma^{2}I\right)}^{-1}\left(\mu -u\right)-\frac{1}{2}\log\left|Q+\sigma^{2}I\right|-\frac{N}{2}\log\left(2\pi \right).$$

We can now use any optimization techniques, e.g., gradient descent, to finish the MAP optimization. Although the fully independent training conditional (FITC) approximation [33] can be used to force $Q_{j}$ to be a diagonal matrix and thus to enable quick computations [32], due to the multiplier $\nabla c^{j}$, Q is generally non-diagonal, and this computation acceleration will not work in our case. The main computation for the joint likelihood (19) is the inverse of joint covariance matrix ${\left(Q+\sigma^{2}I\right)}^{-1}$ and it log determinant $\log|Q+\sigma^{2}I|$. Using an LU decomposition trick [22], we can compute these two terms at time complexity $O(n^{3})$ and space complexity $O(n^{2})$. For the interdiffusion problem, most of the time we have N≤100 EPMA samples, making our method practically efficient.

### 4.6. Interdiffusion Coefficients Predictions

With all model parameters being optimized, we can derive the posterior of the diffusion coefficients for any concentration $c_{*}$ as

(20) $$\begin{array}{cc} d^{j}\left(c_{*}\right) & =N(\mu_{*}^{j},v_{*}^{j}),\\ \mu_{*}^{j} & =\beta_{j}1+{\left(k_{j}^{*}\right)}^{T}{\left(K_{j}\right)}^{-1}\left(h_{j}-\beta_{j}1\right),\\ v_{*}^{j} & ={\left[K_{j}\right]}_{**}-{\left(k_{j}^{*}\right)}^{T}{\left(K_{j}\right)}^{-1}k_{j}^{*}, \end{array}$$

where $k_{j}^{*}={\left(k_{j}\left([c_{*},z_{j1}\right),\cdots ,k_{j}\left(c_{*},z_{jG}\right)\right)}^{T}$ is the covariance between $c_{*}$ and the other ghost coefficient locations $z_{jg}$ and ${\left[K_{j}\right]}_{**}=k_{j}\left(c_{*},c_{*}\right)$. The derivation details are shown in the Appendix A for clarity.

## 5. Results

In practical experiments, the interdiffusion coefficients are unknown and uncontrollable, leading to difficulties for unbiased evaluations. Thus, we first assess InfPolyn on numerical examples of ternary (M=2) and quaternary (M=3) systems. To imitate a real system but not to lose generality, we use polynomial and exponential functions to construct the interdiffusion coefficient functions. To give an example, the fourth-order polynomial function in a two-component system is represented as

(21) $$D_{ij}\left(c^{1},c^{2}\right)=a_{ij}^{0}+\sum_{m=1}^{2}a_{ij}^{m,1}c^{m}+\sum_{m=1}^{2}a_{ij}^{m,2}{\left(c^{m}\right)}^{2}+\sum_{m=1}^{2}a_{ij}^{m,3}{\left(c^{m}\right)}^{3}+\sum_{m=1}^{2}a_{ij}^{m,4}{\left(c^{m}\right)}^{4},$$

where for each coefficient in the polynomial $a_{ij}^{t,r}$, the superscript *r* represents the degree of polynomial and the value of them are generated independently from uniform distributions U(0,1). We put constraints on the high order terms to prevent the diffusion coefficients from increasing/decreasing drastically with the concentrations c; the diffusion matrix is considered symmetric to ensure numerical stability for the diffusion simulations. Note that this symmetric structure prior information is not injected into InfPolyn or other competing models. For the ternary system, the initial conditions for the forward simulation are

(22) $$c^{1}\left(t=0,x\right)=0.6\cdot 𝟙\left(0.5-x\right)$$

(23) $$c^{2}\left(t=0,x\right)=0.4\cdot 𝟙\left(x-0.5\right),$$

where 𝟙(z) denotes the Heaviside step function, which equals to 0 when z<0 and equals to 1 when z≥0. Similarly, for the quaternary system, we defined the initial condition as

(24) $$c^{1}\left(t=0,x\right)=0.6\cdot 𝟙\left(0.5-x\right)$$

(25) $$c^{2}\left(t=0,x\right)=0.25\cdot 𝟙\left(x-0.5\right)$$

(26) $$c^{3}\left(t=0,x\right)=0.15\cdot 𝟙\left(x-0.5\right).$$

With the defined initial condition and the interdiffusion coefficient functions, we use a finite difference (DF) diffusion forward solver to simulate a diffusion process until the terminal time and obtain the terminal concentration profile C. To remain numerically stable and accurate, we use a second-order central difference for space and a fourth-order Runge–Kutta for time. The forward simulation solver uses a spatial step Δx=0.000625, which suggests 1601 grinds points on the space domain $\left[0,1\right]$, the terminal time is set to $10^{4}\;\Delta t$.

We then take equally spaced samples from the terminal concentration profile to mimic the EPMA process to provide the terminal concentration profile C. Unless stated otherwise, the terminal concentration profile consists of 40 samples. Since we are concerned with the center areas where the diffusion process is significant, the EPMA samples are limited in the range of [0.44,0.56] in order to avoid numerical error closed to the boundaries for all Boltzmann–Matano method. All variables are considered dimensionless in the experiments. To evaluate the performance for different methods, we follow Cheng et al. [13] and use the relative error (RE),

(27) $${\mathrm{RE}}_{ij}\left(c\right)=\frac{{\tilde{D}}_{ij}\left(c\right)-D_{ij}\left(c\right)}{D_{ij}\left(c\right)},$$

where ${\tilde{D}}_{ij}\left(c\right)$ and $D_{ij}\left(c\right)$ are the predicted and truth interdiffusion coefficients for concentration c, respectively. As a Boltzmann–Matano numerical inversion-based method, InfPolyn are compared with the other SOTA Boltzmann–Matano numerical inversion-based methods, i.e., the polynomial interdiffusion methods [13] with 3rd and 4th orders of the polynomial, the compress sensing approach [16] combined with 4th-order polynomial (high order model enough to capture the subtle changes), and the $L^{2}$ regularization approach, which replaces the $L^{1}$ penalty term in the work of Qin et al. [16] with an $L^{2}$ penalty term, combined with a 4th-order polynomial function.

### 5.1. Case Study 1: Polynomial Diffusion Coefficients

**Figure 1:**
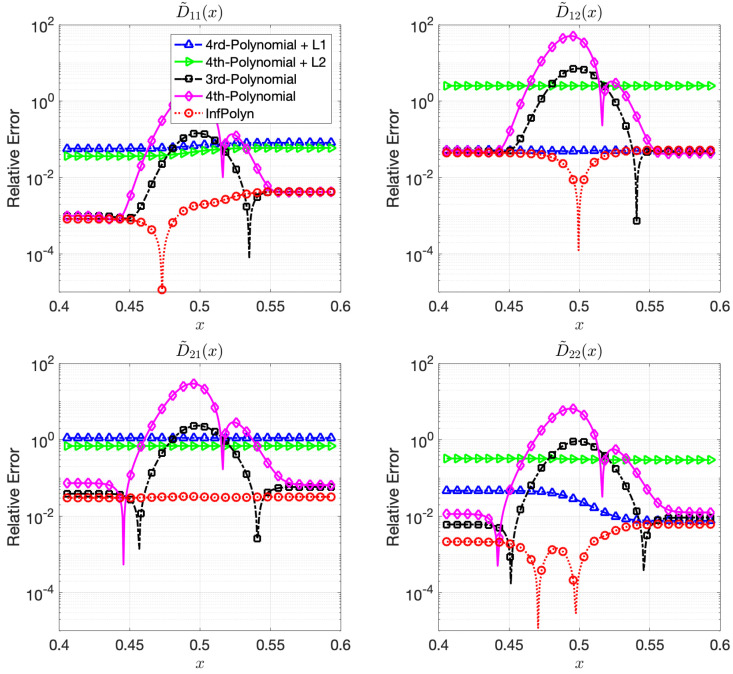
The relative errors (REs) of predictive diffusion coefficients ${\tilde{D}}_{ij}\left(c\left(x\right)\right)$ in the center areas x∈[0.4,0.6] for the evaluated methods in a random ternary system.

**Figure 2:**
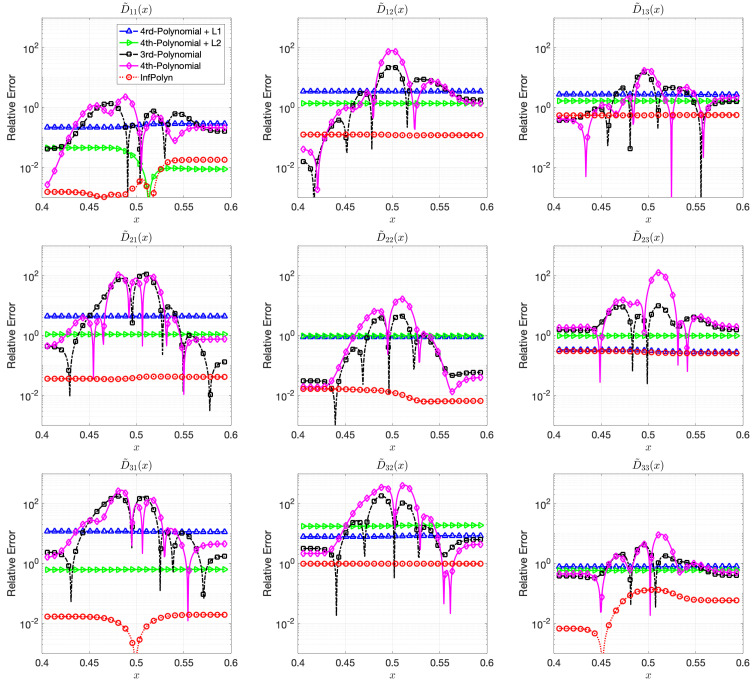
The relative errors (REs) of predictive diffusion coefficients ${\tilde{D}}_{ij}\left(c\left(x\right)\right)$ in the center areas x∈[0.4,0.6] for the evaluated methods in a quadternary system.

In this case study, we assess InfPolyn in a ternary system and a quaternary with 4th-order polynomial interdiffusion coefficients:

(28) $$\begin{array}{c} D_{ij}\left(c\right)=a_{ij}^{m,0}+\sum_{m=1}^{M}\sum_{r=1}^{4}a_{ij}^{m,r}{\left(c^{m}\right)}^{r}, \end{array}$$

where each coefficients $a_{ij}^{m,r}$ are randomly generated using independent uniform distributions. To ensure the symmetrical structure of matrix $A^{m,r}$, we force $a_{ij}^{m,r}=a_{ji}^{m,r}$ by taking their average. In a general interdiffusion process, the interdiffusion coefficients are supposed to be smooth and close to constants, which also prevents instability in the numerical forward solver. To ensure this prior knowledge, we constrain the polynomial coefficients by $a_{ij}^{m,r}∼U\left(0,1\right)10^{\left(r-5\right)}$. The particularly used values are shown in the Appendix A. The REs for x∈[0.4,0.6] for the ternary and the quaternary system are shown in Figure 1 and Figure 2. We omit areas outside [0.4,0.6] because the REs are just extended flat lines without interesting information. As expected, the 4th order polynomial method has a strong model capacity and it can thus achieve few lowest REs at as is shown in some figures within Figure 1 and Figure 2. However, if we look at the whole area of interest, the overall performance is the worst among all methods. In particular, due to the overfitting issue, the 4th order polynomial method shows a highly fluctuational performance, which is highly depreciated for real applications. It is not surprising to learn that the 3rd-order polynomial approach shows slightly fewer fluctuations but also fewer lowest REs. This is indeed the aforementioned dilemma of model selection for the polynomial based methods. Similar to results shown in [16], adding a regularization term of $L^{1}$ can ease the overfitting issue and greatly overcome the performance fluctuation issue in both Figure 1 and Figure 2. Unfortunately, the improvement comes with the price of low model capacity, leading to a rather flat-fitting RE. The 4th-order polynomial method combined with a $L^{2}$ regularization term shows a similar improvement. It is, however, difficult to tell which regularization terms are better. The $L^{1}$ regularization works better with the ternary system in Figure 1, whereas the $L^{2}$ approach outperforms the $L^{1}$ with a large margin in most cases of Figure 2. The inconsistency of performance for the $L^{1}$ and $L^{2}$ regularization approaches certainly hinders their applications for practical problems. In contrast, guided by the correct priors and benefited from the nonparametric nature, InfPolyn shows a consistent and accurate fitting and outperforms the competitors by a significant margin. Thanks to the model flexibility of InfPolyn, it can capture the dramatic changes in the center while maintaining a good fitting in the other flat areas. In all cases, InfPolyn can not only remain stable (indicated by a smooth RE curve) but also achieve the lowest REs in most areas. Furthermore, note that the diagonal interdiffusion coefficients in general show a lower relative error. This is because, in the simulation setting, the diagonal interdiffusion coefficients play a dominant role in the diffusion process. For the non-diagonal interdiffusion coefficients, the REs are amplified by being divided by smaller true interdiffusion coefficients.

### 5.2. Case Study 2: Exponential Diffusion Coefficients

**Figure 3:**
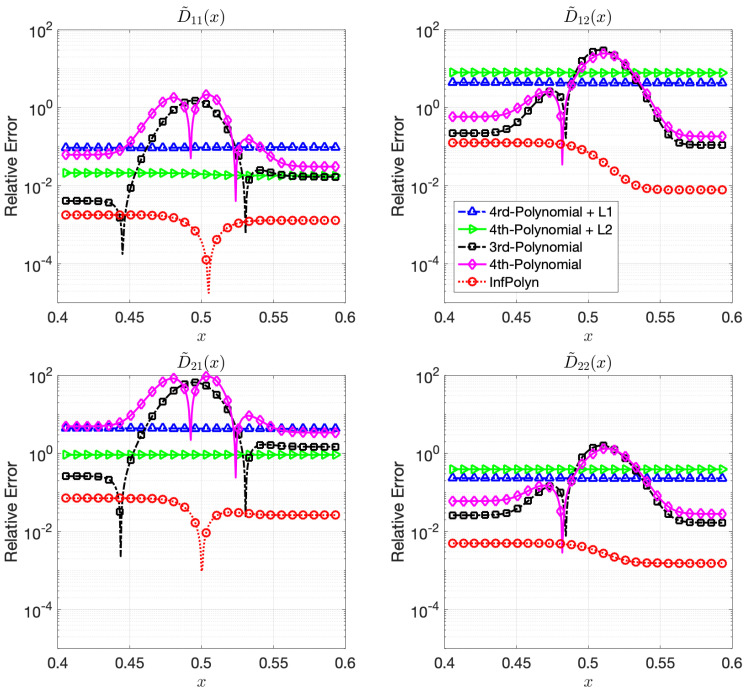
The relative errors (REs) of predictive diffusion coefficients ${\tilde{D}}_{ij}\left(c\left(x\right)\right)$ in the center areas x∈[0.4,0.6] for the evaluated methods in a ternary system.

**Figure 4:**
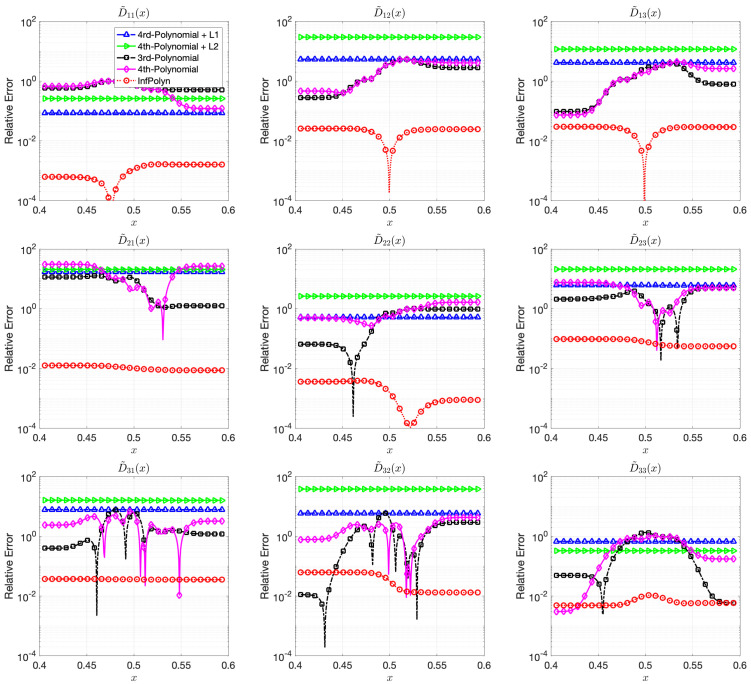
The relative errors (REs) of predictive diffusion coefficients ${\tilde{D}}_{ij}\left(c\left(x\right)\right)$ in the center areas x∈[0.4,0.6] for the evaluated methods in a quadternary system.

In general, the diffusion coefficients can be highly complex that they are not in polynomial forms. To imitate such challenging situations, in this case study, we assess InfPolyn in ternary and quaternary systems with the following interdiffusion coefficient that combines an exponential term and a sinusoidal term,

(29) $$\begin{array}{c} D_{ij}\left(c\right)=a_{ij}^{0}+\sum_{m=1}^{M}a_{ij}^{m,1}\exp\left(-c^{m}\right)-\sum_{m=1}^{M}a_{ij}^{m,2}\cos\left(c^{m}\right), \end{array}$$

where the functional coefficients $a_{ij}^{m,r}$ are similarly sampled from different uniform distributions, i.e., $a_{ij}^{0}∼U\left(0,1\right)\times 10^{-5}$, $a_{ij}^{m,1}∼U\left(0,1\right)\times 10^{-6}$, and $a_{ij}^{m,2}∼U\left(0,1\right)\times 10^{-6}$. Similarly, to ensure the forward diffusion stability, we use the previous approach to ensure the symmetrical structure of matrices $A^{0}$, $A^{m,1}$, and $A^{m,2}$. The used exact values of the functional coefficients are shown in Appendix A. The model performances measured by REs are shown in Figure 3 and Figure 4.

In this case study, the 3rd-order polynomial slightly outperforms the 4th-order polynomial approaches in most cases in both Figure 3 and Figure 4. Nevertheless, the performance of both 3rd-order and 4th-order polynomial approaches are depreciated due to the fluctuation across the domain. Furthermore, note that REs for the polynomial approaches in Figure 4 are flat and smooth, indicating that a rich model capacity does not necessarily lead to performance fluctuations in all cases. The $L^{1}$ and $L^{2}$ regularization combined with 4th-order polynomial degenerate the model performance rather than improving them in many cases in Figure 4. This shows evidence that inappropriate implicit prior assumptions caused by the $L^{1}$ and $L^{2}$ regularization terms can hurt model performance. It might be possible to circumvent this issue by adjusting the penalty weight. However, this will create a new issue of how to properly decide the value of the penalty weight, taking us back to the dilemma of model selections. In contrast, InfPolyn shows a consistent and accurate performance; it outperforms the competitors by a large margin for all cases except for ${\tilde{D}}_{31}$ of the quaternary system in the left area in Figure 4. We would also like to point out that many methods actually fail the quaternary system in Figure 4 as their REs are larger than 1, meaning a total prediction failure.

### 5.3. Case Study 3: Uncertainty Quantification Analysis

**Figure 5:**
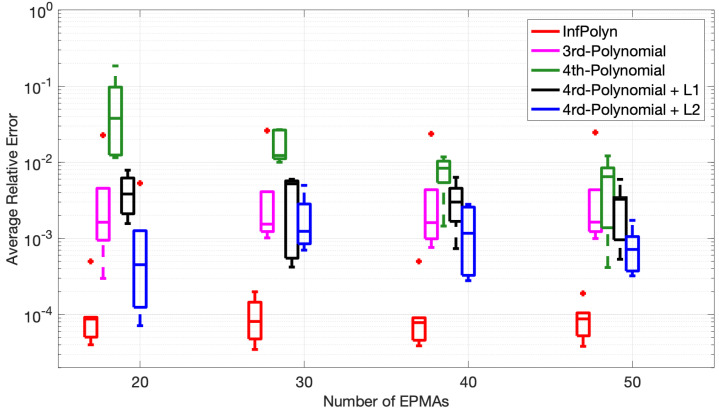

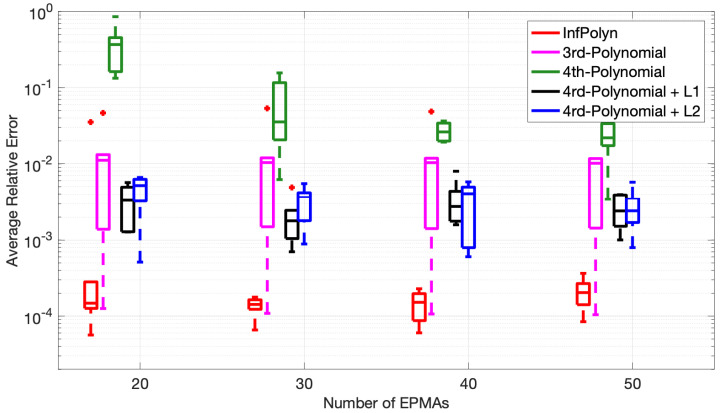
The Tukey box plot of average relative error of ${\tilde{D}}_{11}$ (**top**) and ${\tilde{D}}_{22}$ (**bottom**) based on computation using concentration profile consisting {20,30,40,50} EMPA samples.

Finally, to assess the consistency of InfPolyn, we conducted a ternary system experiment in Case Study 1 based on five distinct random polynomial coefficient sets, which assemble five different diffusion coefficients, and show the performance statistics. To also investigate the influence of the number of the EPMA samples, we ran each experiment with {20,30,40,50} EPMA samples. The minimum number of the EPMA samples was 20 because the 4th-order polynomial has 18 coefficients and thus requires at least 18 EPMA samples to work. For each experiment with the given EPMA samples, the model performance was evaluated by average relative error (ARE),

(30) $${\mathrm{ARE}}_{ij}=\frac{\int_{X}{\mathrm{RE}}_{ij}\left(c\left(x\right)\right)dx}{\int_{X}dx}$$

where X indicates the whole spatial domain. We show the statistics of ${\mathrm{ARE}}_{11}$ and ${\mathrm{ARE}}_{22}$ over the five different diffusion coefficients in Figure 5 using the Tukey box plot. The distinct fact we immediately see is the superiority of InfPolyn compared to the competitors in terms of accuracy and consistency. We then notice that the performance does not improve gradually with the increasing number of EPMA samples for all methods except for the 4th-order polynomial. We believe that each method can already approach reasonable diffusion coefficients (by minimizing the loss function) with only 20 EPMA samples. In this case, more samples will not bring improvement, whereas the performance can fluctuate with different EPMA concentration profiles. Comparing the fluctuations, InfPolyn shows a modest level of changes, whereas the most unstable one is the 4th-order polynomial with $L^{2}$ regularization. The most stable method for both ${\tilde{D}}_{11}$ and ${\tilde{D}}_{22}$ is the 3rd order polynomial, which can indicate a lack of model capacity or an underfitting issue. The only exception of performance improvement is the 4th order polynomial, which improves with more EPMA samples. This is a clear sign of overfitting, which can be addressed by introducing more training data. This explains the overfitting phenomena we previously encountered in Case Studies 1 and 2. Will the performance keep improving and outperform InfPolyn with the trend shown in Figure 5? It may happen with more than 200 EPMA samples, which becomes infeasible in practice. Furthermore, the decreasing trend should slowly disappear at some point, which is already happening for ${\tilde{D}}_{22}$.

It is also noticeable that the $L^{1}$ and $L^{2}$ regularization techniques indeed can improve the performance of a 4th-order polynomial by a large margin for all cases with a different number of EPMA samples, which is consistent with the finding in [16].

### 5.4. Case Study 4: Experiment Verification

**Figure 6:**
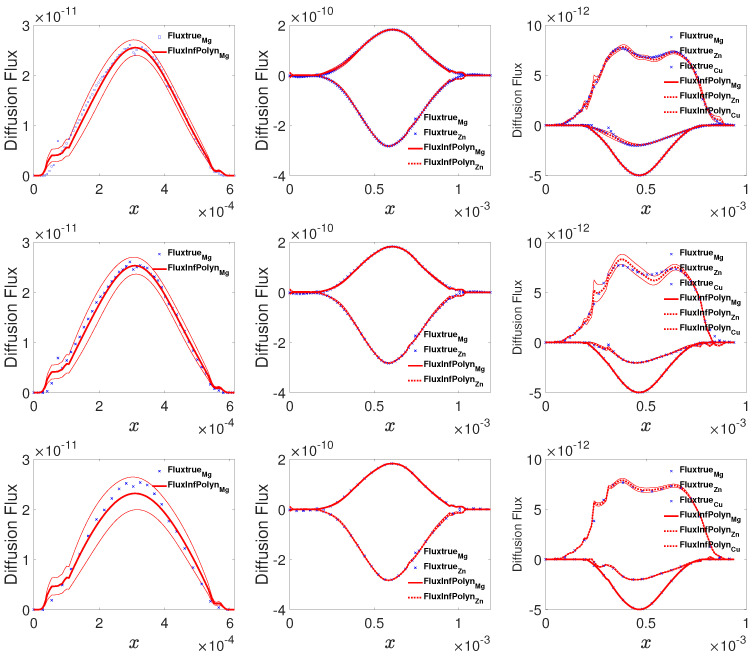
The actual and predicted diffusion fluxes for the Mg-Al, Mg-AL-Zn, and Mg-Al-Zn-Cu system (from left to right columns) using 100%, 50%, and 25% of all available samples (from top to bottom rows).

To present the practical applicability of InfPolyn, we then apply it to the reproduction of the interdiffusion flux from experiment data of the Mg-Al, Mg-Al-Zn, and Mg-Al-Zn-Cu systems collected from the previous literature [13]. These experimental data include composition profiles of the annealed diffusion couples of Mg-Al at 781 K for 36,960 s, Mg-Al-Zn at 868 K for 5400 s, and Mg-Al-Zn-Cu at 755 K for 75,530 s. Since the experimental measurements are taken non-uniformly on the spatial domain for all of the components, they are reprocessed with local polynomials interpolation techniques to provide values on a uniform grid, which is the common preprocessing for the Matano-based approaches. The derivative and integral terms in the Matano equation are then obtained. Given all the preprocessed data as inputs, we then randomly take all of the samples, half of the samples, and a quarter of the samples from the diffusion systems to test the robustness of the testing methods. As shown in Figure 6, the curves for all three cases computed by InfPolyn fit well with the experimental data, which lie in the 95% confident areas, indicating a good uncertainty quantification for the predictions. As for the half size and the quarter size training data, the left areas induce oscillations in some intervals. However, InfPolyn still captures the major tendency of the fluxes with slightly increasing uncertainty.

## 6. Conclusions

In this paper, we propose InfPolyn, a novel nonparametric Bayesian framework to estimate the interdiffusivity coefficients and demonstrate its superiority in terms of accuracy and consistency by combining it with the numerical inverse Boltzmann–Matano method [13]. This also becomes the limitation of InfPolyn because the numerical inverse Boltzmann–Matano method has certain limitations. For instance, it cannot generalize to a wide variety of complicated 2D diffusion processes and complex engineering interdiffusion scenarios, e.g., in semiconductors. Nevertheless, InfPolyn can be combined with other methods (such as the forward-simulation approach [10]) to fulfill its potential in the estimations of interdiffusion coefficients. This is outside the scope of this paper and we thus leave it for the future work.

The main novelty of our work is the nonparametric Bayesian framework that allows automatic model selections (resistant to overfitting and underfitting) and meaningful prior knowledge injections. The problem of recovering interdiffusion predictions from concentrations is an ill-posed inverse problem. Thus, the injection of proper priors is a necessary way to recover the ground-truth diffusion coefficients. Unlike methods such as [16] that impose nonphysical priors, our method provides an easy way to inject intuitive priors; e.g., the diagonal diffusion coefficients normally plays a dominant role in an interdiffusion process.

## Data Availability

All data is in the Appendix A, numerical experiments, and the reference.

## Appendix A. A Gaussian Processs and Its Predicted Posterior

Consider training points $y_{i}=\eta \left(\xi_{i}\right)$, i=1,⋯,M and design points $\xi_{i}$. In a GP model, we place a prior distribution over η(ξ) indexed by ξ:

(A1) $$\eta \left(\xi \right)|\theta ∼GP\left(m\left(\xi \right),c\left(\xi ,\xi^{\prime }|\theta \right)\right)$$

with mean and covariance functions:

(A2) $$\begin{array}{c} m_{0}\left(\xi \right)=E\left[\eta \left(\xi \right)\right],\;c\left(\xi ,\xi^{\prime }|\theta \right)=E\left[\left(\eta \left(\xi \right)-m_{0}\left(\xi \right)\right)\left(\eta \left(\xi^{\prime }\right)-m_{0}\left(\xi^{\prime }\right)\right)\right] \end{array}$$

in which E[·] is the expectation operator. The hyperparameters θ are estimated during the learning process. The mean function can be assumed to be a identical constant, $m_{0}\left(\xi \right)\equiv \mu$, by virtue of centering the data. Alternative choices are possible, e.g., a linear function of ξ, but rarely adopted unless a priori information on the form of the function is available. The covariance function can take many forms, the most common being the automatic relevance determinant (ARD) kernel:

(A3) $$c\left(\xi ,\xi^{\prime }|\theta \right)=\theta_{0}\exp\left(-{\left(\xi -\xi^{\prime }\right)}^{T}\mathrm{diag}\left(\theta_{1},\cdots ,\theta_{l}\right)\left(\xi -\xi^{\prime }\right)\right)$$

The hyperparameters $\theta ={\left(\theta_{0},\cdots ,\theta_{l}\right)}^{T}$. $\theta_{1}^{-1},\cdots ,\theta_{l}^{-1}$ in this case are called the square correlation lengths. For any fixed ξ, η(ξ) is a random variable. A collection of values $\eta (\xi_{i})$, i=1,⋯,M, on the other hand, is a partial realization of the GP. Realizations of the GP are deterministic functions of ξ. The main property of GPs is that the joint distribution of $\eta (\xi_{i})$, i=1,⋯,M, is multivariate Gaussian.

Starting from the prior (A1) and using the available data, we obtain a posterior GP distribution conditional on θ, with new mean and covariance functions. Letting $t={\left(y_{1},\cdots ,y_{M}\right)}^{T}$, the likelihood of the data (given θ) is p(t|θ)=N(μ1,C(θ)). Here, N(·,·) denotes a normal distribution with mean 0 and covariance matrix $C\left(\theta \right)=\left[C_{ij}\right]$, in which $C_{ij}=c\left(\xi_{i},\xi_{j}|\theta \right)$, i,j=1,⋯,M. The joint distribution of t and η(ξ) (for a test input ξ) has the distribution $p\left(\eta \left(\xi \right),t|\theta \right)=N\left(\mu 1,C{\left(\theta \right)}^{\prime }\right)$, where:

(A4) $$\begin{array}{c} C^{\prime }\left(\theta \right)=\left[\begin{array}{cc} C(\theta ) & c(\xi )\\ c{\left(\xi \right)}^{T} & c(\xi ,\xi |\theta ) \end{array}\right] \end{array}$$

in which $c\left(\xi \right)={\left(c\left(\xi_{1},\xi |\theta \right),\ldots ,c\left(\xi_{M},\xi |\theta \right)\right)}^{T}$. Conditioning on t provides the conditional predictive distribution at ξ [22]:

(A5) $$\begin{array}{c} \eta \left(\xi \right)|t,\theta ∼\mathrm{GP}\left(m^{\prime }\left(\xi |\theta \right),c^{\prime }\left(\xi ,\xi^{\prime }|\theta \right)\right)\;\\ m^{\prime }\left(\xi |\theta \right)=\mu 1+c{\left(\xi \right)}^{T}C{\left(\theta \right)}^{-1}\left(t-\mu 1\right)\;\\ c^{\prime }\left(\xi ,\xi^{\prime }|\theta \right)=c\left(\xi ,\xi^{\prime }|\theta \right)-c{\left(\xi \right)}^{T}C{\left(\theta \right)}^{-1}c\left(\xi^{\prime }\right) \end{array}$$

The expected value E[η(ξ)] is given by $m^{\prime }\left(\xi |\theta \right)$, and $c^{\prime }\left(\xi ,\xi |\theta \right)$ is the predictive variance. The hyperparameters θ are normally obtained from point estimates [34,35]. The maximum likelihood estimate (MLE), for example, is found by maximizing the log of the likelihood:

(A6) $$\theta_{MLE}={\arg\max}_{\theta }\left(-\frac{M}{2}\log\left(2\pi \right)-\frac{1}{2}{\left(t-\mu 1\right)}^{T}t^{T}C{\left(\theta \right)}^{-1}\left(t-\mu 1\right)-\frac{1}{2}\ln\left|C\left(\theta \right)\right|\right).$$

### Appendix A.1. Solving a Ternary System Using Boltzmann–Matano Inverse Method

Consider a ternary system where we take EPMA samples at four random locations, $\left[c^{1}\left(x_{n}\right),c^{2}\left(x_{n}\right)\right]$,(n=1,…,4), on the “true” concentration-distance curve at a terminal time. By substituting them into the Boltzmann–Matano equations, we have

(A7) $$\begin{array}{c} \left[\begin{array}{cccc} \;\frac{\partial c_{1}}{\partial x}{|}_{x_{1}} & 0 & \frac{\partial c_{2}}{\partial x}{|}_{x_{1}} & 0\\ \;0 & \frac{\partial c_{1}}{\partial x}{|}_{x_{1}} & 0 & \frac{\partial c_{2}}{\partial x}{|}_{x_{1}}\\ \;\frac{\partial c_{1}}{\partial x}{|}_{x_{2}} & 0 & \frac{\partial c_{2}}{\partial x}{|}_{x_{2}} & 0\\ \;0 & \frac{\partial c_{1}}{\partial x}{|}_{x_{2}} & 0 & \frac{\partial c_{2}}{\partial x}{|}_{x_{2}}\\ \;\frac{\partial c_{1}}{\partial x}{|}_{x_{3}} & 0 & \frac{\partial c_{2}}{\partial x}{|}_{x_{3}} & 0\\ \;0 & \frac{\partial c_{1}}{\partial x}{|}_{x_{3}} & 0 & \frac{\partial c_{2}}{\partial x}{|}_{x_{3}}\\ \;\frac{\partial c_{1}}{\partial x}{|}_{x_{4}} & 0 & \frac{\partial c_{2}}{\partial x}{|}_{x_{4}} & 0\\ \;0 & \frac{\partial c_{1}}{\partial x}{|}_{x_{4}} & 0 & \frac{\partial c_{2}}{\partial x}{|}_{x_{4}} \end{array}\right]\left[\begin{array}{c} D_{11}\\ D_{21}\\ D_{12}\\ D_{22} \end{array}\right]=\left[\begin{array}{c} u_{1}{|}_{x_{1}}\\ u_{2}{|}_{x_{1}}\\ u_{1}{|}_{x_{2}}\\ u_{2}{|}_{x_{2}}\\ u_{1}{|}_{x_{3}}\\ u_{2}{|}_{x_{3}}\\ u_{1}{|}_{x_{4}}\\ u_{2}{|}_{x_{4}} \end{array}\right]. \end{array}$$

We assume second-order polynomials for the functional relationships between diffusion coefficients and concentrations,

(A8) $$\begin{array}{c} \left[\begin{array}{c} D_{11}\\ D_{21}\\ D_{12}\\ D_{22} \end{array}\right]=\left[\begin{array}{c} \alpha_{11}^{\left(0\right)}+\alpha_{11}^{\left(1\right)}c_{1}+\alpha_{11}^{\left(2\right)}c_{2}+\alpha_{11}^{\left(3\right)}c_{1}^{2}+\alpha_{11}^{\left(4\right)}c_{2}^{2}\\ \alpha_{21}^{\left(0\right)}+\alpha_{21}^{\left(1\right)}c_{1}+\alpha_{21}^{\left(2\right)}c_{2}+\alpha_{21}^{\left(3\right)}c_{1}^{2}+\alpha_{21}^{\left(4\right)}c_{2}^{2}\\ \alpha_{12}^{\left(0\right)}+\alpha_{12}^{\left(1\right)}c_{1}+\alpha_{12}^{\left(2\right)}c_{2}+\alpha_{12}^{\left(3\right)}c_{1}^{2}+\alpha_{12}^{\left(4\right)}c_{2}^{2}\\ \alpha_{22}^{\left(0\right)}+\alpha_{22}^{\left(1\right)}c_{1}+\alpha_{22}^{\left(2\right)}c_{2}+\alpha_{22}^{\left(3\right)}c_{1}^{2}+\alpha_{22}^{\left(4\right)}c_{2}^{2} \end{array}\right]=\left[\begin{array}{cc} \phi & 0\\ 0 & \phi \end{array}\right]\left[\begin{array}{c} a_{1}\\ a_{2} \end{array}\right], \end{array}$$

where

(A9)ϕ=Ic1Ic2I(c1)2I(c2)2I(A10)=10c10c20(c1)20(c2)20010c10c20(c1)20(c2)2(A11)ai=α1i(0)α2i(0)α1i(1)α2i(1)α1i(2)α2i(2)α1i(3)α2i(3)α1i(4)α2i(4)T,i=1,2.

We can now simply solve the linear system of equations to obtain the polynomial coefficients (and thus the diffusion coefficients as functions of the polynomials). The procedure is the same with more EPMA samples or higher orders of polynomials. The only difference is that we may solve an overdetermined system with the criteria of minimizing the $L^{2}$ loss.

### Appendix A.2. Experimental Details

Table A1 and Table A2 show the experimental setting of functional coefficients in Case Study 1; Table A3 and Table A4 show the experimental setting of functional coefficients in Case Study 2.

**Table A1:** The polynomial coefficients in the random ternary interdiffusion system, where $a_{ij}$ represents the entries in the coefficient matrix on position {i,j},i=1,2,j=1,2.

A	$a_{11}$	$a_{21}$	$a_{22}$
$A_{0}$	$6.05\times 10^{-5}$	$4.81\times 10^{-6}$	$5.12\times 10^{-5}$
$A_{1}^{1}$	$3.08\times 10^{-6}$	$4.18\times 10^{-7}$	$3.28\times 10^{-6}$
$A_{2}^{1}$	$1.82\times 10^{-6}$	$6.42\times 10^{-7}$	$2.86\times 10^{-6}$
$A_{1}^{2}$	$8.83\times 10^{-7}$	$6.07\times 10^{-8}$	$1.07\times 10^{-7}$
$A_{2}^{2}$	$2.96\times 10^{-7}$	$4.21\times 10^{-8}$	$9.63\times 10^{-7}$
$A_{1}^{3}$	$4.09\times 10^{-8}$	$2.82\times 10^{-9}$	$1.26\times 10^{-8}$
$A_{2}^{3}$	$1.19\times 10^{-8}$	$7.29\times 10^{-9}$	$1.10\times 10^{-8}$
$A_{1}^{4}$	$1.26\times 10^{-8}$	$5.02\times 10^{-10}$	$1.66\times 10^{-9}$
$A_{2}^{4}$	$6.04\times 10^{-9}$	$6.34\times 10^{-10}$	$1.09\times 10^{-8}$

**Table A2:** The polynomial coefficients in the random quaternary interdiffusion system, where $a_{ij}$ represents the entries in the coefficient matrix on position {i,j},i=1,2,3;j=1,2,3.

A	$a_{11}$	$a_{12}$	$a_{13}$	$a_{22}$	$a_{23}$	$a_{33}$
$A_{0}$	$2.03\times 10^{-5}$	$6.50\times 10^{-6}$	$5.15\times 10^{-6}$	$7.13\times 10^{-5}$	$3.58\times 10^{-7}$	$3.27\times 10^{-5}$
$A_{1}^{1}$	$9.15\times 10^{-6}$	$3.13\times 10^{-7}$	$6.87\times 10^{-7}$	$9.46\times 10^{-6}$	$1.35\times 10^{-7}$	$1.76\times 10^{-6}$
$A_{2}^{1}$	$9.56\times 10^{-6}$	$8.08\times 10^{-7}$	$3.01\times 10^{-7}$	$1.61\times 10^{-6}$	$4.89\times 10^{-7}$	$1.18\times 10^{-6}$
$A_{3}^{1}$	$2.01\times 10^{-6}$	$9.05\times 10^{-7}$	$2.47\times 10^{-7}$	$5.48\times 10^{-6}$	$7.41\times 10^{-7}$	$2.95\times 10^{-6}$
$A_{1}^{2}$	$2.61\times 10^{-7}$	$7.28\times 10^{-8}$	$6.95\times 10^{-8}$	$2.18\times 10^{-7}$	$5.91\times 10^{-8}$	$1.58\times 10^{-7}$
$A_{2}^{2}$	$5.90\times 10^{-7}$	$2.23\times 10^{-8}$	$4.55\times 10^{-8}$	$7.08\times 10^{-7}$	$5.79\times 10^{-8}$	$3.94\times 10^{-7}$
$A_{3}^{2}$	$2.17\times 10^{-9}$	$2.96\times 10^{-8}$	$5.60\times 10^{-8}$	$8.11\times 10^{-7}$	$3.82\times 10^{-8}$	$6.70\times 10^{-8}$
$A_{1}^{3}$	$9.00\times 10^{-8}$	$2.45\times 10^{-9}$	$2.52\times 10^{-10}$	$9.00\times 10^{-8}$	$5.90\times 10^{-9}$	$2.48\times 10^{-8}$
$A_{2}^{3}$	$2.70\times 10^{-8}$	$2.57\times 10^{-9}$	$2.11\times 10^{-9}$	$6.50\times 10^{-10}$	$6.48\times 10^{-9}$	$6.01\times 10^{-9}$
$A_{3}^{3}$	$8.04\times 10^{-8}$	$2.55\times 10^{-9}$	$3.11\times 10^{-9}$	$8.91\times 10^{-8}$	$2.76\times 10^{-9}$	$1.03\times 10^{-8}$
$A_{1}^{4}$	$4.82\times 10^{-9}$	$3.18\times 10^{-10}$	$1.87\times 10^{-10}$	$9.07\times 10^{-9}$	$2.45\times 10^{-10}$	$3.91\times 10^{-9}$
$A_{2}^{4}$	$6.58\times 10^{-9}$	$9.28\times 10^{-10}$	$2.44\times 10^{-10}$	$2.05\times 10^{-9}$	$6.07\times 10^{-10}$	$7.94\times 10^{-9}$
$A_{3}^{4}$	$9.38\times 10^{-9}$	$1.30\times 10^{-10}$	$9.50\times 10^{-10}$	$9.17\times 10^{-9}$	$6.05\times 10^{-10}$	$1.46\times 10^{-9}$

**Table A3:** The table shows the setting for coefficient matrix of polynomials function and $A_{i}\;and\;A_{i}^{{}^{\prime }}$ represents the entries in each coefficient matrix on position {i},i=1,2;j=1,2.

A	$a_{11}$	$a_{21}$	$a_{22}$
$A_{0}$	$6.15\times 10^{-5}$	$5.08\times 10^{-7}$	$5.46\times 10^{-5}$
$A_{1}$	$1.25\times 10^{-7}$	$3.77\times 10^{-7}$	$8.50\times 10^{-8}$
$A_{2}$	$9.09\times 10^{-7}$	$3.51\times 10^{-7}$	$9.08\times 10^{-7}$
$A_{1}^{{}^{\prime }}$	$4.85\times 10^{-7}$	$2.85\times 10^{-8}$	$7.39\times 10^{-7}$
$A_{2}^{{}^{\prime }}$	$4.67\times 10^{-7}$	$7.29\times 10^{-7}$	$8.27\times 10^{-7}$

**Table A4:** The table shows the setting for coefficient matrix of exponential function and $A_{i}\;and\;A_{i}^{{}^{\prime }}$ represents the entries in each coefficient matrix on position {i},i=1,2,,3;j=1,2,3.

A	$a_{11}$	$a_{12}$	$a_{13}$	$a_{22}$	$a_{23}$	$a_{33}$
$A_{0}$	$4.82\times 10^{-5}$	$4.61\times 10^{-6}$	$4.17\times 10^{-6}$	$4.26\times 10^{-5}$	$4.34\times 10^{-6}$	$6.27\times 10^{-5}$
$A_{1}$	$8.77\times 10^{-8}$	$8.40\times 10^{-8}$	$1.07\times 10^{-8}$	$9.65\times 10^{-8}$	$8.20\times 10^{-8}$	$2.62\times 10^{-8}$
$A_{2}$	$5.15\times 10^{-8}$	$8.39\times 10^{-9}$	$1.87\times 10^{-8}$	$9.39\times 10^{-8}$	$1.55\times 10^{-8}$	$9.78\times 10^{-8}$
$A_{3}$	$6.81\times 10^{-8}$	$2.67\times 10^{-8}$	$2.23\times 10^{-8}$	$7.68\times 10^{-9}$	$5.41\times 10^{-9}$	$3.78\times 10^{-8}$
$A_{1}^{{}^{\prime }}$	$3.45\times 10^{-8}$	$3.79\times 10^{-8}$	$3.48\times 10^{-8}$	$3.11\times 10^{-9}$	$1.42\times 10^{-8}$	$5.00\times 10^{-8}$
$A_{2}^{{}^{\prime }}$	$1.21\times 10^{-9}$	$3.53\times 10^{-8}$	$6.64\times 10^{-8}$	$7.51\times 10^{-8}$	$2.52\times 10^{-8}$	$5.45\times 10^{-8}$
$A_{3}^{{}^{\prime }}$	$6.21\times 10^{-8}$	$3.77\times 10^{-8}$	$6.93\times 10^{-8}$	$1.23\times 10^{-9}$	$6.45\times 10^{-8}$	$1.75\times 10^{-8}$
